# Manufacturing and Analysis of Overmolded Hybrid Fiber Polyamide 6 Composite

**DOI:** 10.3390/polym13213820

**Published:** 2021-11-04

**Authors:** Heru S. B. Rochardjo, Cahyo Budiyantoro

**Affiliations:** 1 Department Mechanical and Industrial Engineering, Universitas Gadjah Mada, Yogyakarta 55281, Indonesia; 2 Department of Mechanical Engineering, Universitas Muhammadiyah Yogyakarta, Bantul 55183, Indonesia; cahyo_budi@umy.ac.id

**Keywords:** polyamide 6, hybrid fiber, overmolding, cryogenic treatment, Box–Behnken design, flexural strength, impact strength, ILSS

## Abstract

Currently, fiber-reinforced thermoplastic composites are widely applied in structural applications. It has great potential to replace metal structures and provides advantages in weight reduction. In this study, the pretensioned unidirectional carbon fiber was overmolded by Polyamide 6 contained 30%wt of glass fibers (PA 6-30GF). Process parameters such as injection pressure, melting temperature, duration of carbon fiber cryogenic treatment, and fiber pretension were optimized to maximize the flexural strength, impact strength, and interlaminar properties of the hybrid composite. The relationship between factors and responses was analyzed using Box–Behnken design (BBD) from response surface methodology (RSM) and analysis of variance (ANOVA). Three levels were assigned for each factor. There were 27 experimental trials carried out, and a significant regression for the coefficient between the factors was derived. The BBD and ANOVA analysis demonstrate that the predicted values from the model are in satisfactory correlation with the experimental results. The optimum responses found were achieved by setting the following injection molding parameters: melting temperature of 278 °C and injection pressure of 122 bar. Carbon fiber, as a unidirectional reinforcement, should be immersed in liquid nitrogen for 10 min and mounted on the mold in a pretensioned state with a force of 100 N. The combination of these parameters can produce an optimum flexural strength of 248.6 Mpa, impact strength of 173.4 kJ/m^2^ and an ILSS of 30.4 Mpa.

## 1. Introduction

Composites for structural applications can be made by combining short and continuous fibers and/or combining different types of fibers. Both glass and carbon fibers are common synthetic fibers used as reinforcements, embedded in a thermoplastic matrix. The glass fibers are relatively cheap, easily obtained, have high a strength-to-weight ratio, but low stiffness. Comparing to glass fiber, carbon fiber is more expensive and has lower compressive strength, but the strength-to-weight ratio and stiffness are higher. Carbon fibers also have lower density and better thermal and electrical conductivity than glass fibers. Due to the ease and speed of their production process, short fibers are preferred as reinforcement over continuous fibers [1]. Short fibers perform well in resisting impact loads but are less efficient at supporting flexural and tensile loads. Tensile and flexural loads are the domains of continuous fiber reinforcement. For structural composites with a combination of loading conditions, hybridization of discontinuous fibers and continuous fibers reinforcement is required [2,3]. Moreover, hybridization of those two types of fibers was aimed to reduce the weaknesses of both and to get the advantages of each of them.

In automotive applications, the combination of flexural and impact loads often occurs in several components, for example in bumpers. The use of composite materials for these components needs to consider the ease and speed of the process as well as low production costs. The injection overmolding is one of the production methods that can be developed to produce hybrid fiber-reinforced thermoplastic composites. With this method, one can combine various types of fibers in one composite product. The quality of the injection product is influenced by the setting of process parameters. Process parameters can affect the physical performance of the composite in the form of geometry and dimensions, fiber length after filling, fiber orientation, and the fiber volume fraction in the resulting composite [4]. The parameters can be optimized to improve mechanical properties [5]. The process parameters that can be controlled include injection pressure, cylinder heating temperature, packing pressure and packing time, cooling temperature and time, injection speed, and back pressure [6]. Setting A high injection pressure and high melting temperature can facilitate the flow of plastic melt-carrying fibers into the mold cavity. The appropriate injection pressure ensures the physical quality of the product with precise dimensions and geometries that are free of sink marks and voids. However, a too-high injection pressure can result in fiber damage. The melting temperature is normally adjusted according to the type of matrix material and follows the recommendations of the material manufacturer. High melting temperatures decrease the viscosity of the plastic melt so that they penetrate the fiber more easily; however, too-high melt temperatures can degrade the matrix. Optimizing processing parameters is an essential issue in obtaining high flexural and impact properties for short fiber-continuous fiber hybrid composite products. In general, the hybrid injection-overmolding process involves using thermoforming sheets as the core of the product [7]. Some plastic-material manufacturing industries, such as BASF and Lanxess, and injection-machine manufacturing industries, such as Engel and KraussMaffei, have developed technological solutions for injection molding-based hybrid composites. However, using unidirectional fiber directly as the core of the specimen still has the potential to be developed [8].

The adhesion between fiber and matrix is also a factor that determines the quality of structural composites. Cryogenic fiber treatment is a practical, effective, and environmentally friendly method to enhance the bonding strength between carbon fiber and polymer matrix. Shao et al. [9] immersed the carbon fiber in liquid nitrogen at −196 °C, this treatment increased the adhesive strength of the carbon fiber/epoxy composite by up to 31%. Cryogenic treatment basically cannot change the chemical composition of the fiber; only morphological changes occur in the form of an increase in surface roughness. Song et al. [10] stated that increasing the surface roughness can improve the surface energy of the fiber.

Structural strength analysis of composites containing continuous fibers often uses the assumption that the fibers are in a straight state, while in fact, the condition of wavy fibers can occur. The assumption of the straight condition can cause over-prediction in determining the compressive properties of unidirectional fibrous composites. Fiber tension control is applied to the filament winding and pultrusion processes. In the process of manufacturing composite structural composites using the injection molding method, fiber pretension has not been carried out. The stress that occurs during mechanical property testing is affected by fiber tension during the process. In general, an increase in tension will increase the strength of the component if the fiber dominates the load bearing [11,12].

This study uses the injection-overmolding technique in the manufacture of hybrid fiber reinforced polyamide-6 composites. The composite is composed of pretensioned uni-directional carbon fiber and overmolded by polyamide 6 containing 30% glass fibers. The injection process parameters were varied to show the sensitivity of PA 6 GF on melting temperature and injection pressure. Cryogenic treatment was applied to unidirectional carbon fiber before overmolding. The parameter factors were divided into three levels: low, medium, and high. The target of this research is to determine the effect of process parameters on the flexural strength and the impact strength of composite and the inter-laminar shear strength (ILSS) between fiber and matrix in the composite system. The data analysis used the Response Surface Method-Box Behnken Design and ANOVA.

## 2. Experimental

### 2.1. Materials

The carbon fibers T700SC 12K, made by Toray, Tokyo, Japan, were used as unidirectional reinforcement [13]. The Amilan PA 6-30GF produced by Toray Resins Europe GmbH, Germany, was used as unidirectional reinforcement. The material is a polyamide (PA) 6 that contains 30% glass fiber, a type of engineering plastic with high ductility and impact resistance [14]. The properties of the materials involved in this study are shown in Table 1. PA 6 easily absorbs moisture, therefore drying needs to be done by using a hot-air dryer. Drying is recommended at 80–120 °C for 6 h until the humidity content is around 0.15 to 0.2%.

### 2.2. Manufacturing of Hybrid Overmolded Composites

**Figure 1:**
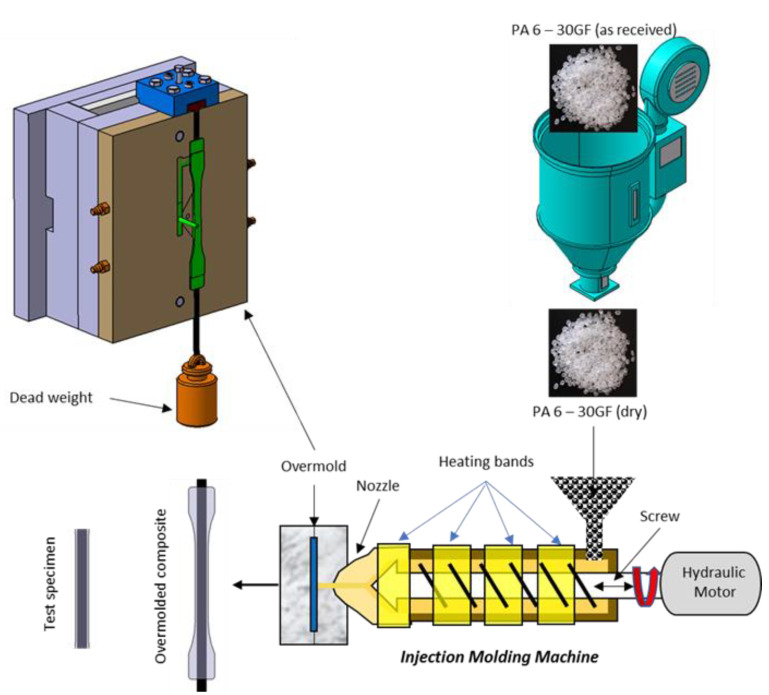
Manufacturing steps of hybrid fiber overmolded composite.

The manufacturing stages of the hybrid fiber overmolded composite are shown in Figure 1. As a unidirectional reinforcement, the carbon fiber was cut along the mold length, mounted on a mold equipped with a pre-tension device. One end of the fiber was clamped on the chuck, while the other end was pulled with varying tension: 20%, 30%, and 40% of the ultimate strength of fiber, respectively. The pretension is obtained by static loading, since the cross-section area of the carbon fiber bundle is 7 mm × 7 μm = 0.049 mm^2^, and the load applied to the fiber was 50 N, 75 N, and 100 N respectively. The deadweight method was applied to the fiber pretension, that is, simply applying a constant weight to the unidirectional fibers before plastic melt injection [15]. The load was released after the plastic reaches the solidification state. Before being attached to the mold, the carbon fiber was immersed in liquid nitrogen for three time periods of 10, 15, and 20 min. This treatment aimed to increase the surface roughness of fibers so that the mechanical interlocking between the matrix and fibers can be increased. Immersing carbon fiber in liquid nitrogen for 10 min has been proven to produce better fiber-matrix adhesion of CFRP than silane coupling agents [16]. A FANUC ROBOSHOT -S-2000i100A injection machine with a clamping capacity of 100 tons was used to produce overmolding specimen A. PA 6-GF pellet was fed through the hopper of the injection molding machine, plasticized, and transported to the nozzle. The melted matrix containing short glass fibers was overmolded onto pretensioned unidirectional fibers. Two injection parameters were varied: injection pressure and melting temperature. The hydraulic injection pressure was varied by 100 bar, 120 bar, and 140 bar. The melting temperature was adjusted as follows: 260 °C, 270 °C, and 280 °C [17], following material manufacturer recommendation. Other injection process parameters were set constantly, according to the initial trial results and recommendations from the matrix material supplier, as shown in Table 2.

### 2.3. Design of Experiment

Response surface methodology (RSM) is an effective method for linking factors to the expected response. This is a statistically based method that has many advantages, including the capability for evaluating interactions between variables, less experimental trial, easy-to-understand graphical data display, investigated all factor-level combinations, and it can develop mathematical models that connecting factors and responses [18,19]. The mathematical model can be expressed as a regression model as described in Equation (1):

(1) $$Y=\beta_{0}+\overset{k}{\underset{i=1}{\sum }}\beta_{i}X_{i}+\overset{k}{\underset{i=1}{\sum }}\beta_{ii}X_{i}^{2}+\overset{k-1}{\underset{i=1}{\sum }}0\overset{k}{\underset{i>j,j=2}{\sum }}\beta_{ij}X_{i}X_{j}+\epsilon$$

where *Y* is the predicted response related to the model, and $\beta_{0}$ corresponds to constant, $\beta_{i}$, $\beta_{ii}$, and $\beta_{ij}$ are the linear, quadratic, and interaction coefficients, *X* is process factor, and *k* is the number of the factors [20,21].

In this study, the Box–Behnken design (BBD), a type of RSM design, was used to determine the effect of process factors on the target responses. There were 4 factors with each divided into three levels (low, middle, and high) used in this study, as shown in Table 3. Melting temperature and injection pressure are controllable injection parameters that govern the flow behavior of the matrix containing short fiber. Fiber pretensions influence the role of unidirectional fiber, while nitrogen immersion affects the fiber-matrix adhesion. These factors and levels are arranged in the experimental design as shown in Table 4. The experimental design was carried out with 27 trials and in each trial, 5 repetitions were carried out. The trials consisted of 24 cube points and 3 central points at run 1, 4, and 20. Flexural strength, impact strength, and ILSS were the target responses of this experiment.

### 2.4. Characterization

The specimens were subjected to three types of mechanical properties characterization: a flexural test, an impact test, and interlaminar shear strength (ILSS).

A three-point- bending test was performed, according to ISO 178, on the samples’ flexural properties using a Zwick/Roell Z20 Proline universal test machine. The test was performed at a displacement rate of 2 mm/min [22]. The testing specimen had a thickness of 5.5 mm, a width of 10 mm, and a length of 110 mm (20 times of thickness). According to the standard, the span length was 88 mm, 16 times the thickness of the specimen [22].

The interlaminar shear strength (ILSS) of the composite was evaluated by short beam shear tests following ASTM D-2344 [23]. Dumbbell specimens were cut into a rectangular specimen of dimension 40 mm × 10 mm × 5.5 mm. At room temperature, the tests were carried out on a GOTECH universal testing machine with a crosshead speed of 2 mm/min. A three-point bending device equipped with 3-mm-diameter supports and a 6-mm-diameter indenter nose was adjusted to a span of 20 mm. The interlaminar shear strength (ILSS) can be calculated according to Equation (2).

(2) $$ILSS=0.75\frac{F_{m}}{w\cdot t}$$

where:

*ILSS* = interlaminar shear strength, Mpa

*F_m_* = maximum load recorded during the test, N

*w* = specimen width, mm

*t* = specimen thickness, mm

At least five specimens per set of parameter combinations were tested.

The impact tests were carried out on the GOTECH testing machine using the Charpy unnotched flatwise method following ISO 179 [24]. The width and thickness of the test specimen were 10 mm and 5.5 mm, respectively. Impact strength was calculated based on the initial angle of the pendulum hammer and the final angle after impact, as show in in Equations (3) and (4) [24].

(3) $$E_{c}=W\times R(\cos\beta -\cos\alpha )$$

(4) $$a_{c}=\frac{E_{c}}{h\cdot b}\times 10^{3}$$

where:

- *W* = pendulum weight.
- *R* = pendulum arm length, m.
- *A* = initial angle of the pendulum hammer.
- *Β* = final angle after impact.
- *E_c_* = corrected energy, absorbed by breaking test specimen, J.
- *a_c_* = Charpy impact strength, kJ/m^2^
- *h* = specimen thickness, mm.
- *b* = specimen width, mm.

All these three tests were performed at a temperature of 23 °C and relative humidity (RH) of approximately 50%. For each trial, at least five identical samples were tested, and average values and standard deviations were recorded.

## 3. Results and Discussion

### 3.1. Testing Results

**Figure 2:**
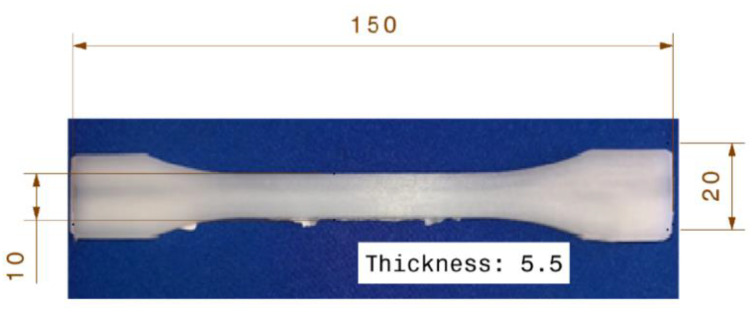
Result of hybrid fiber overmolded composite.

Table 5 shows the test results on the overmolding specimens, the values of flexural strength, impact strength, and ILSS in the table are the average values of five measurements from each run. The manufacturing of overmolded hybrid fiber composite produced a multipurpose specimen, as shown in Figure 2.

The results of 27 trials showed that the highest flexural strength of 252.8 Mpa was obtained from run 5 with a combination of parameters: melting temperature of 280 °C, the injection pressure of 120 bar, fiber pretension of 75 N, and fiber immersion for 10 min. Run 21, with a combination of parameters—melting temperature of 270 °C, the pressure of 140 bar, fiber pretension of 50 N, and fiber immersion for 15 min—resulted in the highest impact strength value of 173.4 kJ/m^2^. The highest ILSS results were obtained in run 1 with a combination of parameters: melting temperature 270 °C, an injection pressure of 120 bar, fiber pretension of 75 N, and fiber immersion for 15 min. The interesting thing is that even the lowest flexural strength and impact values from the experiment were higher than the PA 6 30 GF values. The lowest flexural strength result was given by the 18th run, of 207.2 Mpa, an increase of about 42% compared to the raw matrix. The lowest impact strength result occurred in the third run, of 137.3 kJ/m^2^, 37% higher than the raw matrix. Meanwhile, the lowest ILSS was seen on the 24th run, of 16.4 MPa. The lowest flexural strength result was given by the 18th run of 207.2 Mpa, an increase of about 42% compared to the raw matrix. The lowest impact strength results occurred in the third run of 137.3 kJ/m^2^, 37% higher than the raw matrix. Meanwhile, the lowest ILSS was seen on the 24th run of 16.4 MPa. According to Maier et al. [25], the ILSS of PA6-CF is in the range of 21 to 28 Mpa at room temperature. This shows the contribution of carbon fiber as a unidirectional reinforcement. However, because the design of experiment did not represent all combinations of factors and levels, further analysis was needed to find opportunities for higher response values and to analyze the significance of each factor on the response.

### 3.2. Analysis of Variance (ANOVA) and Model Fitting of the Responses

The experimental results were analyzed using F-test-based ANOVA. The ANOVA can be used to evaluate the relationship between responses and process factors. The significance of factors on the three target responses can be observed.

#### 3.2.1. Flexural Strength

Table 6 shows the results of ANOVA for flexural strength, in this case, the mathematical model follows a linear order. From the functional analysis of the relationship between factors and flexural strength, it is known that the function fits a first-order polynomial regression model, with a *p*-value of 0.0323; this reflects the overall fit of the model. If this case uses a second-order polynomial, a *p*-value of 0.157 is obtained, which is greater than the specified significant level (α = 0.05).

The Model F-value of 3.21 demonstrates that the model was significant. There is only a 3.23% chance that an F-value of this amount could occur due to noise. Values of “Prob > F” less than 0.0500 indicate that model’s terms are significant. In this situation, A model (melt temperature) is a significant model term. The “Lack of Fit F-value” of 0.75 indicates that the Lack of Fit is not significant in comparison to the pure error. A non-significant lack of fit is preferable since it shows that the model fits the response. “Adeq Precision” calculates the signal-to-noise ratio; a ratio greater than four is preferable. The ratio of 5.79 indicates an adequate signal, and the model can be used to navigate the design space. The relationship between the model-forming factors and the flexural strength response, in terms of actual factors, can be expressed as written in the Equation (5):

Flexural strength = +44.019 + 0.636 × A + 0.2 × B + 0.097 × C − 0.69 × D(5)

where A is the melt temperature, B is the injection pressure, C is the fiber pretension, and D is the duration of carbon fiber immersion in liquid nitrogen. This equation can be interpreted as all factors except nitrogen immersion time having a positive effect on flexural strength. Here, melting temperature has the most significant effect on flexural strength because it can reduce the viscosity of the matrix so that the glass fiber can be evenly distributed. The negative effect of immersion time means that a longer immersion time of unidirectional carbon fiber in liquid nitrogen results in lower flexural strength.

#### 3.2.2. Impact Strength

Table 7 describes the ANOVA for impact strength, second-order polynomial can be used as a mathematical model for this response. For the impact strength response, the second-order polynomial gives a *p*-value of 0.043 (significant model), while the first-order polynomial gives a *p*-value of 0.694 (not significant model).

The Model F-value of 3.82 proves the model was significant. There is only a 1.27% chance that an F-value of this amount could occur due to noise. Interaction of melt temperature (A) and fiber pretension (C), and fiber pretension, in quadratic order, significantly influenced the impact strength. “Adeq Precision” is 7.55, which indicates an adequate signal, so this model can be used to navigate the design space. Equation (6) is the mathematical model derived from the ANOVA, in terms of actual factors:

(6) Impact strength = −2035.69 + 20.85 A − 2.74 B − 15.44 C + 13.32 D + (3.33 × 10^−3^) AB + 0.052 AC − 0.036 AD + (2.6 × 10^−3^) BC − 0.027 BD − 0.039 CD − 0.045 A^2^ + 9.24 B^2^ + 9.89 C^2^ + 0.088 D^2^

where linear factors melt temperature and fiber immersion had a positive effect, while the injection pressure and fiber pretension had a negative effect, on the impact strength. High-temperature application on pretensioned fiber helps the unidirectional fiber maintain its condition and experience better relaxation.

#### 3.2.3. Interlaminar Shear Strength (ILSS)

Table 8 shows the ANOVA results for ILSS. The Model F-value of 3.61 indicates the model is significant. There is only a 1.59% chance that an F-value this large could occur due to noise. In this case, A, B, AD, A^2^, B^2^, and C^2^ are significant model terms. A value greater than 0.1000 indicates that the model terms are insignificant. It can be seen that all factors contribute to ILSS. The higher injection temperature and pressure make it easier for the melted matrix to carry the short glass fibers into the mold and penetrate the pretensioned carbon fibers. The interaction of a low matrix viscosity with an appropriate immersion time can facilitate such penetration.

Here “Adeq Precision” of 6.867 is adequate, so the model can be applied to navigate the design space. The mathematical model in terms of actual factors is shown in Equation (7).

(7) $$\begin{array}{c} \mathrm{ILSS}=-4245.35+29.67\;A+0.057\;B+1.75\;C+24.43\;D+(7.4\times 10^{-3})\mathrm{AB}-\\ 1.61\;\mathrm{AC}-0.0798\;\mathrm{AD}\;–\;(2.675\times 10^{-3})\mathrm{BC}\;–\;(6.75\times 10^{-3})\mathrm{BD}-0.0186\;\mathrm{CD}-\\ 0.0538\;A^{2}\;–\;(7.735\times 10^{-3})B^{2}\;–\;(4.712\times 10^{-3})\;C^{2}-0.026\;D^{2} \end{array}$$

### 3.3. Diagnostic Plots of the Responses

**Figure 3:**
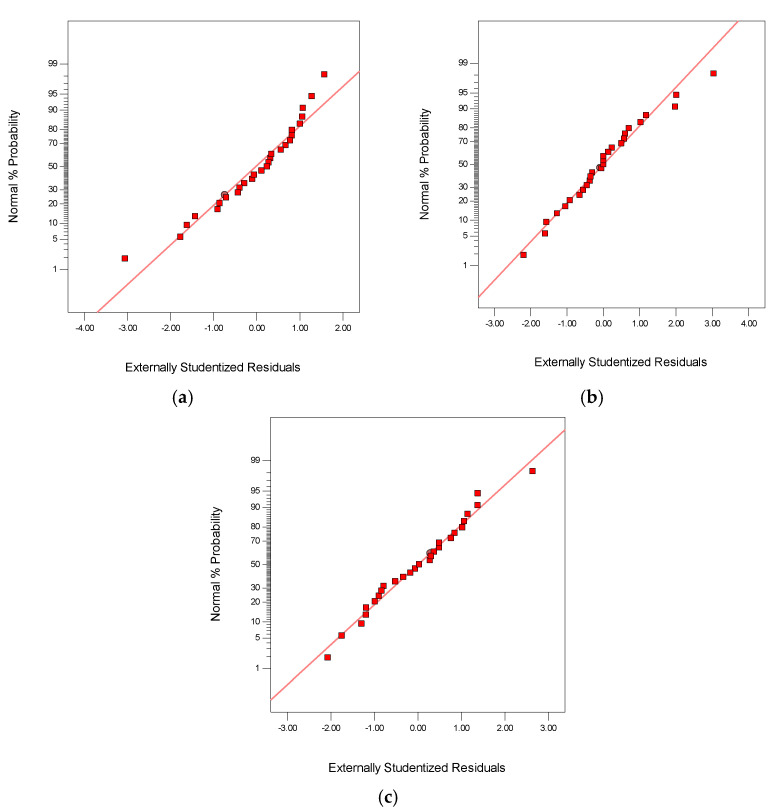
Normal probability plot residuals for the responses: (**a**) flexural strength; (**b**) impact strength; (**c**) ILSS.

An examination of the suitability of the model with the real system is necessary to ensure that the model provides an adequate estimate. The normal probability plot is one of the diagnostic tools to provide an estimate of the adequacy of the model [26]. Figure 3 display the normal probability plot of the residuals for the flexural strength, impact strength, and ILSS, respectively. All of the residuals plots are close to the ideal line of regression, proving that the errors are normally distributed.

### 3.4. Effect of Process Factors on the Responses

**Figure 4:**
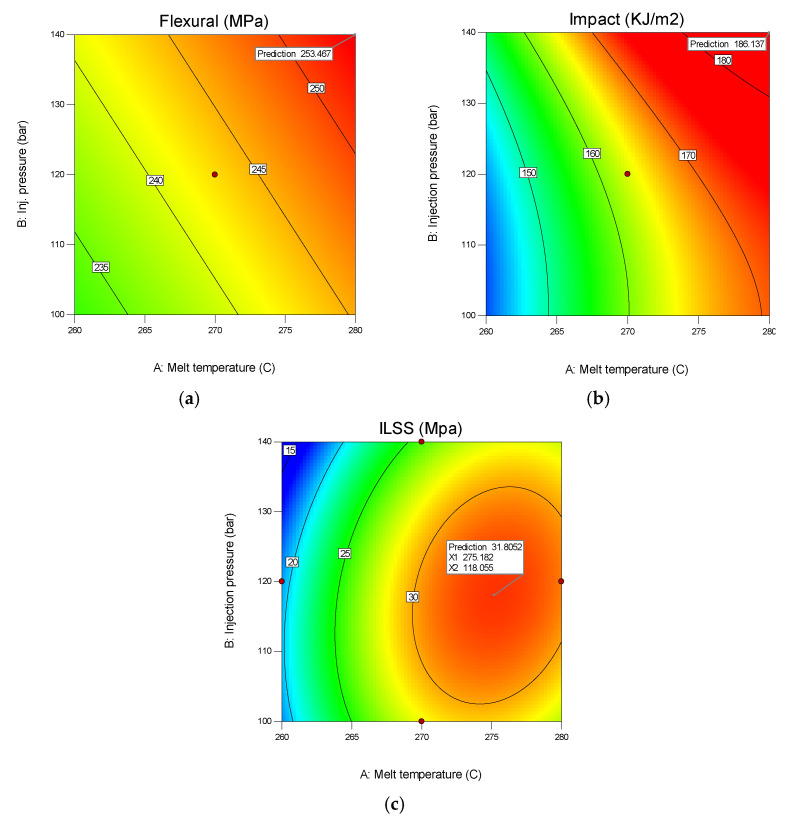
Effect of injection pressure and melt temperature on the target responses: (**a**) flexural strength; (**b**) impact strength; (**c**) ILSS.

Figure 4 shows the effect of the process factor on the target responses. The model predicts that the highest flexural strength of 253.47 Mpa can be achieved by a combination of the following process parameters: melting temperature 280 °C, injection pressure 140 bar, fiber pretension 100 N, and immersion time of 10 min. The highest predicted impact strength is 186,137 kJ/m^2^, this value is achieved by the same combination as mentioned previously. It means that both mechanical properties can be obtained by setting the injection process parameters and fiber pretension at the maximum value, while the immersion time of the fiber is set for 10 min. Longer immersion time does not have a positive effect on mechanical properties.

In contrast to the two mechanical properties above, the highest ILSS prediction of 31.8 Mpa was produced by a combination of process parameters: melting temperature 275.18 °C, injection pressure 118 bar, fiber pretension 75 N, and immersion time 10 min. Immersion time for 10 min is proven to contribute to high responses value.

### 3.5. Optimization of Process Parameters

**Figure 5:**
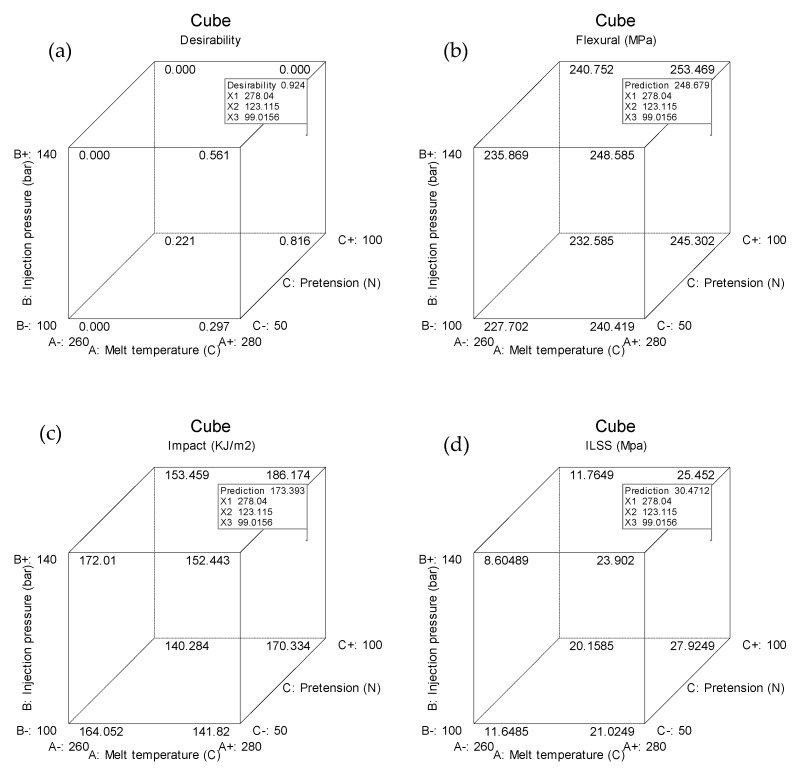
Optimization results: (**a**) desirability; (**b**) flexural strength; (**c**) impact strength; (**d**) ILSS.

In the previous step, a combination of parameters has been obtained to maximize the value of each response. Maximum flexural strength and impact strength were obtained with the same process parameters, but not for ILSS. Optimization needs to be done to get a combination that can maximize all responses, known as multi-objective optimization [27,28,29]. A desirability function can be used to optimize multiple responses at the same time. Figure 5 shows the results of Box–Behnken design optimization. The optimal process parameters for all three responses are found to be the melt temperature of 278.06 °C, the injection pressure of 122.97 bar, a fiber pretension of 99 N, and an immersion time of 10 min. With these parameters, the optimal responses generated are as follows: flexural strength of 248.67 Mpa, impact strength of 173.4 kJ/m^2^, and ILSS of 30.47 Mpa. The desirability of this optimization result is 0.924, close to one. Desirability is an objective function with a value between zero and one. The value zero is assigned to factors that produce an undesirable response, whereas the value one denotes the optimal condition for the observed factors. The conditions with the highest desirability value are considered as the best values for the targeted response [30,31]. To maximize the desirability function of each response, the following Equation can be used [32]:

(8) $$d=\{\begin{array}{c} {\left|\frac{y-LSL}{USL-LSL}\right|}^{s},\;\;\;\;LSL<y<USL\;\\ 0,\;\;\;\;\;\;\;\;\;\;\;\;\;\;\;\;\;\;\;\;\;\;\;\;\;\;\;\;\;\;\;\;\;\;\;\;\;\;y<LSL\\ 1,\;\;\;\;\;\;\;\;\;\;\;\;\;\;\;\;\;\;\;\;\;\;\;\;\;\;\;\;\;\;\;\;\;\;\;\;\;\;y>USL \end{array}$$

where the exponent *s* is the shape constant of the desirability function and *LSL* and *USL* are the lower and upper response limits, respectively. Equation (9) can be used to calculate the overall desirability, *D*. Below, *d_f_*, *d_i_*, and *d_s_* are the desirability functions of flexural strength, impact strength, and ILSS, respectively.

(9) D=(df×di×ds)13

The optimization results can be proposed as a systematic multiobjective model for the injection overmolding process. It produces consistent parametric factors with multiobjective target responses, with less quality variation, and maintains product quality within an acceptable range.

### 3.6. Confirmation Experiment

The confirmation experiments were carried out using the combination of parameters that resulted from the optimization stage. Two experiments were carried out to validate the proposed optimization model using the following parameter combinations: melt temperature of 278 °C, the injection pressure of 122 bar, fiber pretension of 100 N, and fiber immersion time for 10 min. The process variables and corresponding yields for each experiment are shown in Table 9. 

The experimental value obtained was reasonably close to the predicted value derived from the model, as shown in the table above. Here, the percentage errors for the confirmation experiment values and the predicted performance values at optimal process conditions were less than 3% for all responses. It is proven that Box Behnken Design and ANOVA are powerful predictive tools to determine the combination of process parameters for optimum multiple responses.

### 3.7. Microstructure of Fractured Surface

**Figure 6:**
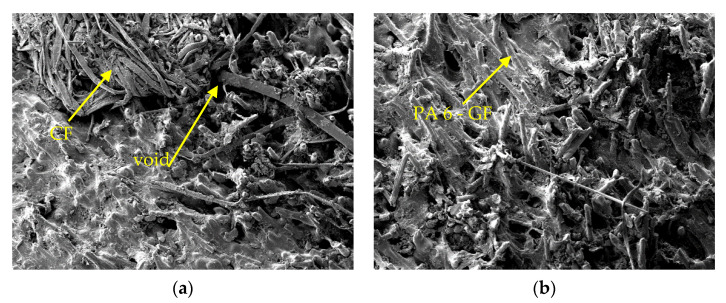
Micrograph of fracture surface: (**a**) low impact resistance specimen; (**b**) high impact resistance specimen.

Figure 6a shows the micrograph of the fracture surface resulting from the low resistance impact test specimen. In contrast, Figure 6b displays the fracture surface of high resistance impact resistance. Glass fibers appear to bond perfectly with the PA 6 matrix because they have been incorporated in the form of composite pellets as a commercially manufactured material. Through comparing the two micrographs it can be stated that the lower impact resistance is related to the poor adhesion between unidirectional carbon fiber and the matrix. Significant gaps were found in the contact region between carbon fiber and PA 6, indicating that the interfacial bond between carbon fiber and PA 6 is much lower than between glass fiber and PA 6. In Figure 6a, there are some visible voids and agglomerated carbon fibers. This condition is due to the application of low pretension to carbon fiber combined with a low melting temperature setting. The carbon fiber cannot withstand the injection pressure, and the plastic melt cannot enter the gaps between the carbon fibers.

Different conditions are shown in Figure 6b; the voids and carbon fiber agglomeration are less than Figure 6a. The high melting temperature can reduce the viscosity of the plastic melt so that it is easier to flow into the gaps between the UD carbon fibers. The application of high pretension on carbon fiber can increase the stability of the fiber against the injection pressure of molten plastic and reduce the tendency to agglomerate. That situation is also the reason for the good mechanical performance.

## 4. Conclusions

The Box–Behnken design and ANOVAs have successfully demonstrated the effect of the injection overmolding process parameters on the mechanical properties of the hybrid fiber reinforced PA 6. Melting temperature had the most significant effect on flexural strength, while the interaction of melt temperature and fiber pretension, and fiber pretension in quadratic order, significantly influenced the impact strength. ILSS was significantly affected by melt temperature, injection pressure, the interaction of melt temperature and fiber immersion time, and the quadratic order of melt temperature, injection pressure, and fiber pretension. Immersing unidirectional carbon fiber for 10 min in liquid nitrogen conferred a positive effect to all three composite properties. To achieve the optimum composite mechanical properties, it is recommended to use a melting temperature of 278 °C, an injection pressure of 122 bar, a fiber pretension of 100 N, and to immerse the carbon fibers for 10 min in liquid nitrogen.

## Figures and Tables

**Table 1:** Material properties.

Material	Properties	Values
carbon fiber (T700SC 12K)	filament diameter (µm)	7
	density (g/cm^3^)	1.8
	tensile strength (Mpa)	4900
Amilan PA 6-30 GF	density (g/cm^3^)	1.36
	melting temperature (°C)	225
	flexural strength at 23 °C (Mpa)	280 (dry)
		145 (2.5% water)
	flexural modulus at 23 °C (Gpa)	9.5 (dry)
		5.1 (2.5% water)
	charpy impact, unnotched, at 23 °C (kJ/m^2^)	80 (dry)
		100 (2.5% water)
liquid nitrogen	boiling point (°C)	−196
	density, liquid @ BP, 1 atm (Kg/m^3^)	808.5
	specific gravity, liquid (water = 1) @ 20 °C, 1 atm	0.808

**Table 2:** Constant parameter setting.

Parameters	Values	Unit
screw speed	160	rpm
charging time	3.42	s
backpressure	50	bar
hot size	70	mm
velocity transfer pressure	35	mm
injection speed	80	mm/s
filling time	0.535	s
packing pressure	300	kg/cm^2^
packing time	2	s
cooling time	15	s
cooling temperature	40	°C

**Table 3:** Parameter factors and levels.

Factors	Coding	Actual Level		
		Low (−1)	Middle (0)	High (+1)
Melting temperature (°C)	A	260	270	280
Injection pressure (bar)	B	100	120	140
Fiber pretension (N)	C	50	75	100
Nitrogen immersion (min)	D	10	15	20

**Table 4:** Design of experiment.

Run	Code				Actual			
	A	B	C	D	A	B	C	D
1	0	0	0	0	270	120	75	15
2	1	0	−1	0	280	120	50	15
3	−1	0	1	0	260	120	100	15
4	0	0	0	0	270	120	75	15
5	1	0	0	−1	280	120	75	10
6	0	0	1	−1	270	120	100	10
7	−1	0	0	−1	260	120	75	10
8	1	1	0	0	280	140	75	15
9	0	1	0	−1	270	140	75	10
10	1	−1	0	0	280	100	75	15
11	−1	0	0	1	260	120	75	20
12	0	−1	0	−1	270	100	75	10
13	0	−1	1	0	270	100	100	15
14	0	0	1	1	270	120	100	20
15	0	0	−1	−1	270	120	50	10
16	1	0	0	1	280	120	75	20
17	0	0	−1	1	270	120	50	20
18	−1	−1	0	0	260	100	75	15
19	−1	0	−1	0	260	120	50	15
20	0	0	0	0	270	120	75	15
21	0	1	−1	0	270	140	50	15
22	0	1	0	1	270	140	75	20
23	1	0	1	0	280	120	100	15
24	−1	1	0	0	260	140	75	15
25	0	−1	0	1	270	100	75	20
26	0	−1	−1	0	270	100	50	15
27	0	1	1	0	270	140	100	15

**Table 5:** Testing results.

Run	Parameters						
	A	B	C	D	Flexural Strength (Mpa)	Impact Strength (kJ/m^2^)	ILSS (Mpa)
1	270	120	75	15	245.4	153.271	32.6
2	280	120	50	15	238	144.569	24.53
3	260	120	100	15	241.2	137.285	19.36
4	270	120	75	15	247.5	153.271	29.9
5	280	120	75	10	252.8	151.469	30
6	270	120	100	10	249.2	166.974	28.85
7	260	120	75	10	245.6	151.621	20.97
8	280	140	75	15	250	155.148	26.8607
9	270	140	75	10	246.8	166.879	24
10	280	100	75	15	238.6	151.469	24.86
11	260	120	75	20	235.2	158.979	26.8
12	270	100	75	10	233.2	148.862	29.9
13	270	100	100	15	235	155.225	30.2
14	270	120	100	20	241.2	157.775	24.48
15	270	120	50	10	239	153.271	24.83
16	280	120	75	20	237.8	151.545	19.86
17	270	120	50	20	237.4	162.506	29.8
18	260	100	75	15	207.2	147.942	20.32
19	260	120	50	15	221.5	166.974	21.63
20	270	120	75	15	230	153.271	28.4
21	270	140	50	15	233.4	173.393	21.28
22	270	140	75	20	242	162.28	21
23	280	120	100	15	234.2	167.162	20.65
24	260	140	75	15	224.4	148.957	16.4
25	270	100	75	20	231.4	155.072	29.6
26	270	100	50	15	233.2	171.479	22.72
27	270	140	100	15	231	162.356	23.41

**Table 6:** ANOVA for flexural strength.

Source	Sum of Squares	df	Mean Square	F-Value	*p*-Value Prob > F	Note
Model	900.98	4	225.24	3.21	0.0323	significant
A-Melt temperature	485.14	1	485.14	6.90	0.0154	significant
B-Injection pressure	200.08	1	200.08	2.85	0.1056	
C-Fiber pretension	71.54	1	71.54	1.02	0.3239	
D-Nitrogen immersion	144.21	1	144.21	2.05	0.1660	
Residual	1545.90	22	70.27			
Lack of Fit	1363.30	20	68.16	0.75	0.7155	not significant
Pure Error	182.61	2	91.30			
Cor Total	2446.88	26				
Adeq Precision	5.79					

**Table 7:** ANOVA for impact strength.

Source	Sum of Squares	df	Mean Square	F-Value	*p*-Value Prob > F	Note
Model	1558.28	14	111.31	3.82	0.0127	significant
A-Melt temperature	7.69	1	7.69	0.26	0.6168	
B-Injection pressure	126.52	1	126.52	4.34	0.0592	
C-Fiber pretension	53.83	1	53.83	1.85	0.1991	
D-Nitrogen immersion	6.87	1	6.87	0.24	0.6359	
AB	1.78	1	1.78	0.061	0.8092	
AC	683.34	1	683.34	23.45	0.0004	significant
AD	13.25	1	13.25	0.45	0.5128	
BC	6.80	1	6.80	0.23	0.6376	
BD	29.21	1	29.21	1.00	0.3364	
CD	84.96	1	84.96	2.92	0.1134	
A^2^	110.17	1	110.17	3.78	0.0756	
B^2^	72.92	1	72.92	2.50	0.1396	
C^2^	204.11	1	204.11	7.01	0.0213	significant
D^2^	26.27	1	26.27	0.90	0.3611	
Residual	349.61	12	29.13			
Lack of Fit	349.61	10	34.96			
Pure Error	0.000	2	0.000			
Cor Total	1907.90	26				
Adeq Precision	7.55					

**Table 8:** ANOVA for ILSS.

Source	Sum of Squares	df	Mean Square	F-Value	*p*-Value Prob > F	Note
Model	383.63	14	27.40	3.61	0.0159	significant
A-Melt temperature	37.74	1	37.74	4.97	0.0456	significant
B-Injection pressure	50.63	1	50.63	6.67	0.0240	significant
C-Fiber pretension	0.39	1	0.39	0.051	0.8247	
D-Nitrogen immersion	4.10	1	4.10	0.54	0.4767	
AB	8.76	1	8.76	1.15	0.3037	
AC	0.65	1	0.65	0.085	0.7751	
AD	63.76	1	63.76	8.40	0.0134	significant
BC	7.16	1	7.16	0.94	0.3507	
BD	1.82	1	1.82	0.24	0.6329	
CD	21.81	1	21.81	2.87	0.1158	
A^2^	154.53	1	154.53	20.36	0.0007	significant
B^2^	51.06	1	51.06	6.73	0.0235	significant
C^2^	46.27	1	46.27	6.10	0.0295	significant
D^2^	2.27	1	2.27	0.30	0.5949	
Residual	91.08	12	7.59			
Lack of Fit	82.02	10	8.20	1.81	0.4078	not significant
Pure Error	9.06	2	4.53			
Cor Total	474.70	26				
Adeq Precision	6.867					

**Table 9:** Results of model validation at the optimum condition.

Entry	Predicted Flexural Strength	Confirmation Flexular Strength	Predicted Impact Strength	Confirmation Impact Strength	Predicted ILSS	Confirmation ILSS
	(Mpa)	(Mpa)	(kJ/m^2^)	(kJ/m^2^)	(Mpa)	(Mpa)
1	248.67	249	173.4	171.4	30.47	30.26
2		246		169.5		29.99
